# Fabrication of Hollow and Porous Tin-Doped Indium Oxide Nanofibers and Microtubes via a Gas Jet Fiber Spinning Process

**DOI:** 10.3390/ma13071539

**Published:** 2020-03-27

**Authors:** Monoj Ghosh, Sadhan C. Jana

**Affiliations:** Department of Polymer Engineering, The University of Akron, 250, South Forge Street, Akron, OH 44325–0301, USA; mg84@zips.uakron.edu

**Keywords:** ITO, hollow fibers, porous nanofibers, microtubes, gas jet spinning

## Abstract

We report the morphologies of tin-doped indium oxide (ITO) hollow microtubes and porous nanofibers produced from precursor solutions of polyvinylpyrrolidone (PVP), indium chloride (InCl_3_), and stannic chloride (SnCl_4_). The polymer precursor fibers are produced via a facile gas jet fiber (GJF) spinning process and subsequently calcined to produce ITO materials. The morphology shows strong dependence on heating rate in calcination step. Solid porous ITO nanofibers result from slow heating rates while hollow tubular ITO microfibers with porous shells are produced at high heating rates when calcined at a peak temperature of 700 °C. The mechanisms of formation of different morphological forms are proposed. The ITO fibers are characterized using several microscopy tools and thermogravimetric analysis. The concentration of inorganic salts in precursor solution is identified as a key factor in determining the porosity of the shell in hollow fibers. The data presented in this paper show that GJF method may be suitable for fabrication of hollow and multi-tubular metal oxide nanofibers from other inorganic precursor materials.

## 1. Introduction

One dimensional (1-D) nanostructures of inorganic oxides namely nanofibers [1,2,3,4,5,6,7], nanorods [6], nanotubes [7], or nanowires [8] present unique optical, electronic, magnetic, sensor, and opto-electronic properties, and are suitable for many applications. Among these, hollow inorganic nanostructures enjoy special attention in adsorption [9], sensors and devices [10,11,12], photo-electrochemical cells [13,14], photocatalysis [14], and filtration at high temperatures and under corrosive environment [15], all due to their hollow structure and accessibility to both interior and exterior surface area and stability at high temperature. To date, a number of methods have emerged reporting the synthesis of inorganic oxide nanotubes or nanofibers, namely template synthesis [16], co-axial electrospinning techniques [17], microfluidic compound jet electrospinning [18], anodization technique [19], and hydrothermal method [20]. The above methods produce hollow or mesoporous inorganic oxide nano/micro tubes or fibers. 

Zhang et al. [14] synthesized hollow TiO_2_ nanofibers with surface area as high as 118 m^2^ g^−1^ using a two-step synthesis approach consisting of electrospinning of titanium isopropoxide (Ti(O*i*Pr)_4_) with two immiscible polymers polyethylene oxide (PEO) and polyvinylpyrrolidone (PVP) using a core-shell needle followed by template removal via annealing at 450 °C. The idea of co-axial electrospinning technique was utilized later for the fabrication of hollow TiO_2_ nanostructure from a PVP solution containing Ti(O*i*Pr)_4_ and mineral oil, followed by selective removal of the liquid core and calcination [17]. 

A few other studies [2,7,21] reported one step direct traditional electrospinning route followed by thermal treatment for fabrication of tubular micro or nanofibers. Lang et al. [2,7] reported fabrication of porous Fe_2_O_3_ microtubes and tube-in-tube nanofibers by controlling the heating rate during calcination process of electrospun precursor fibers. Recently, Wu et al. [21] studied the mechanism of SnO_2_ nanotube fabrication by direct electrospinning and calcination of PVP-SnCl_2_ composite fibers. They showed structural evolution from dense fibers to wire-in-tube to nanotubes of SnO_2_ during thermal decomposition of fibers containing PVP-SnCl_2_. These authors proposed PVP-assisted Ostwald ripening as the mechanism for SnO_2_ nanotube structure formation. The above method does not require an additional template removal step or the use of a co-axial needle design for fabrication of hollow structure nanofibers or tubes. It is noted that all the hollow fibrous materials obtained in earlier studies were produced primarily from core-shell [17] or traditional electrospinning technique [2,5,13] with diameter ranging from a few tens of nanometer to a few micrometers. It is also noted that electrospinning process requires high voltage and is often associated with low production rate per nozzle. 

A new scalable fiber spinning process, called the gas jet fiber (GJF) spinning process [22,23,24], was reported recently to meet the demands of moderate to large quantities of non-woven micro/nanofibers while not utilizing electrical potential. The GJF process is known to produce polymer nanofibers at substantially higher rates in comparison to single nozzle electrospinning [24]. The GJF process was used recently in fabrication of semiconducting metal oxide (SMO) micro and nanofibers from polymer precursors, such as ITO-TiO_2_ core-shell and side-by-side morphology [25] using two different nozzle designs or mesoporous TiO_2_ nanofibers [26] and hierarchical V_2_O_5_ nanorods on TiO_2_ nanofibers [6]. The GJF process can produce SMO fibers of diameter ranging from ~50 to 550 nm at a rate ~30 times higher than that of single nozzle electrospinning jet [23]. The SMO nanofibers showed much enhanced ethanol photocatalytic oxidation activities [6,26]. The strategy of fabrication of hollow inorganic oxide fibers with porous shells using the GJF spinning process is reported for the first time in this paper. 

Tin-doped indium oxide (ITO) is an important multifunctional metal oxide in view of its growing number of applications in light-emitting devices (LEDs) [27], dye sensitized solar cell [28,29,30], as anodic material for Li ion batteries [31], photovoltaic [32], in sensors [33], conductive and transparent surfaces [34], plasma and touchscreen displays [35], to name a few. Some reports on the fabrication of 1-D nanofibrous morphology of ITO based on electrospinning in combination with sol-gel chemistry were recently published for the above-mentioned applications [5,28,30,33,34,36,37]. However, the porous hollow ITO micro/nanofibers were not reported in the literature to the best of our knowledge. 

This paper presents a controllable one-step process for fabrication of porous hollow or solid tubular micro- and hollow nanofibers from ITO. A possible formation mechanism of different structures of ITO is also highlighted. The paper presents results on the effects of ITO precursor concentration on morphology development under controllable heating rate during calcination. The morphology of ITO fibers and tubes were characterized by field emission scanning electron microscopy (FESEM) and transmission electron microscopy (TEM). The crystalline phase structure and chemical composition of the fibers were confirmed using X-ray diffraction (XRD) and energy dispersive X-ray (EDS) methods.

## 2. Materials and Methods 

Polyvinylpyrrolidone (PVP, Average Mw~1,300,000 g/g mol, Sigma Aldrich, St. Louis, MO, USA) was used as received. Indium chloride (InCl_3_, 4H_2_O) (>97% purity) and tin chloride (SnCl_4_, 5H_2_O) (Sigma Aldrich, USA) were used as inexpensive precursor materials for the fabrication of ITO fibers. A mixed solvent of ethanol (EtOH) (>99.5% pure) (Fischer Scientific, Hampton, NH, USA) and N, N-dimethyl formamide (DMF) (Sigma Aldrich, USA) was used for the preparation of PVP-InCl_3_-SnCl_4_ sol solution for spinning. These chemicals were used without further purification.

### 2.1. Preparation of Spinning Sol Solution: PVP-InCl_3_-SnCl_4_

In a typical procedure, 1 g PVP was dissolved in 8 mL of mixed solvent of EtOH/DMF (6/4) (v/v) % by magnetically stirring at room temperature for ~30 minutes in order to obtain 10.0 (w/w)% PVP solution. In the next step, specific quantities of salts InCl_3_.4H_2_O and SnCl_4_.5H_2_O were dissolved in 4 mL of the mixed solvent (EtOH/DMF) maintaining a 90:10 (w/w)% of In:Sn in the solution. A small amount of deionized (DI) water in each recipe was added to make a clear solution of the salts. The amount of ITO precursor salts in final spinning sol solution was varied from 4.5 (w/w)% to 18.0 (w/w)% of the solution weight in the following steps: 4.5, 9.0, 12.0, and 18.0 (w/w)%. This allowed investigation of the effects of precursor concentration on ITO fiber morphology. The spinning sol solution was obtained by mixing ITO precursor solution with previously prepared 10.0 (w/w)% PVP solution in a Thinky^®^ mixer (THINKY USA, Laguna Hills, CA, USA) under airtight condition for ~20 minutes at room temperature. 

### 2.2. Fabrication of ITO Fibers

**Figure 1:**
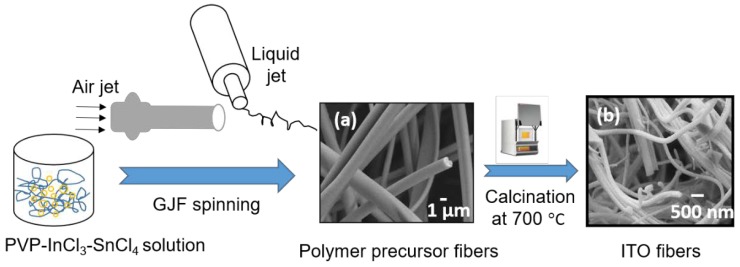
Schematic diagram of a needle-tip GJF spinning process showing ITO fibers fabrication process and morphologies of (**a**) PVP-InCl_3_-SnCl_4_ salt precursor fibers from 9.0 (w/w)% ITO precursor loaded spinning sol solution, and (**b**) corresponding ITO nanofibers after calcination at 700 °C with a heating rate of 0.5 °C min^−1^.

The spinning sol solution containing PVP, InCl_3_, and SnCl_4_ was taken into a 10 mL HSW plastic syringe, which was linked with a 25-gauge needle JG17-0.5X, (Jensen Global, CA, USA) of internal diameter Ø = 0.89 mm. The sol solution was injected through the needle using a syringe pump (Model-Fusion 200). The sol solution injection rate was 0.3 mL min^−1^ unless otherwise stated. House air at a pressure of 20 psi and a volumetric air flow rate of 0.1339 standard cubic feet per minute was used in the GJF process. Air was injected through a nozzle of inner diameter of 1.2 cm. The distance between the nozzle delivering air and the needle-tip was 1.5 cm. A detailed description of the GJF spinning process was presented elsewhere [22,23]. A piece of flat nylon sieve was placed at 180–200 cm from the tip of the needle to collect the polymer precursor fibers containing PVP, InCl_3_, and SnCl_4_. The GJF spinning set up was placed horizontal and the spinning took place under ambient condition of temperature (~25 ± 2 °C) and relative humidity (~25%–35%). A schematic presented in Figure 1 shows the steps involved in making ITO fibers.

The collected fibers were kept in air for ~2 h at room temperature and ~25%–35% relative humidity for hydrolysis of metal chloride salts. The precursor fibers were then calcined in air at a maximum temperature of 700 °C using a muffle furnace (Model: Vulcan 3–130). Calcination occurred in several stages at two heating rates of 0.5 °C min^−1^ (slow) and 5 °C min^−1^ (high). First, the temperature was increased from room temperature to 100 °C at the specified heating rate and kept at 100 °C for 1 h. Second, the temperature was raised to 700 °C at the same heating rate and maintained at 700 °C for 2 h. At this stage, the traces of organic components in the fibers were removed, leaving ITO crystals organized in the form of fibers. Finally, the material was cooled naturally to room temperature. 

### 2.3. Materials Characterization

The surface topography and morphology of the GJF-spun fibers before and after calcination were investigated by field emission scanning electron microscopy (FESEM, JEOL JSM-7401F, JEOL USA, Peabody, MA, USA) and transmission electron microscopy (TEM, JEOL JSM-1230, JEOL USA). The samples were sputter coated with silver using high resolution ion beam sputtering system model ISI 5400 under argon atmosphere. The FESEM images were taken using JEOL JSM-7401F at an accelerating voltage of 5–10 kV, emission currents of 10–20 mA, and magnifications in the range 500–80,000X. High quality FESEM images were used in measurement of fiber diameter distributions in conjunction with processing software, Image J, NIH, USA. The diameter of more than 100 fibers were measured from multiple of FESEM images. A straight line was drawn along the diagonal of an image to ensure that fibers were not counted twice and the diameter was measured if the fibers crossed the line. 

Energy dispersive X-ray spectroscopy (EDS, GENESIS4000, EDAX, Mahwah, NJ, USA) attached to FESEM was used to determine the elemental composition of the polymer precursor fibers and the resultant ITO fibers obtained by calcination. TEM was used to study the crystalline surface morphology and the grain size of the calcined nanofibers. Thermogravimetric analysis (TGA) (Q200 from TA Instruments, New Castle, DE, USA) was carried out at a heating rate of 10 °C min^−1^ from room temperature to 700 °C using air flow of 60 mL min^−1^ to determine the thermal stability and calcination conditions of the polymer precursor fibers. Powder X-ray diffraction (Bruker) (Ultima IV, X-Ray Diffractometer, Rigaku, Woodlands, TX, USA) with Cu-K radiation (λ = 0.15418 nm) of prepared ITO fibers was carried out to obtain crystallographic information and phase structure in each sample. The viscosity of solutions of PVP and the precursor sol solutions was measured over a range of shear rate, 1 to 500 s^−1^, under steady state condition at 27 °C using Advance Rheometric Expansion System (ARES) Rheometer (Model: G2 from TA Instruments) fitted with a concentric cylindrical fixture.

## 3. Results and Discussion

### 3.1. Morphology and Crystal Structure of ITO Micro/Nano Fiber

**Figure 2:**
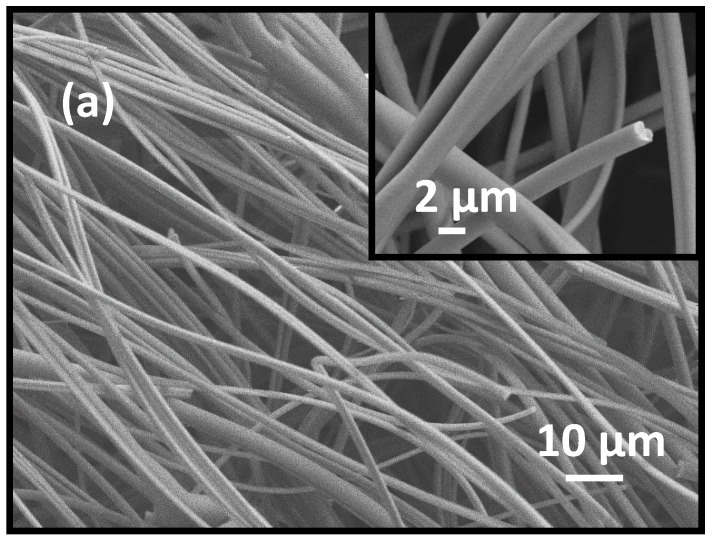

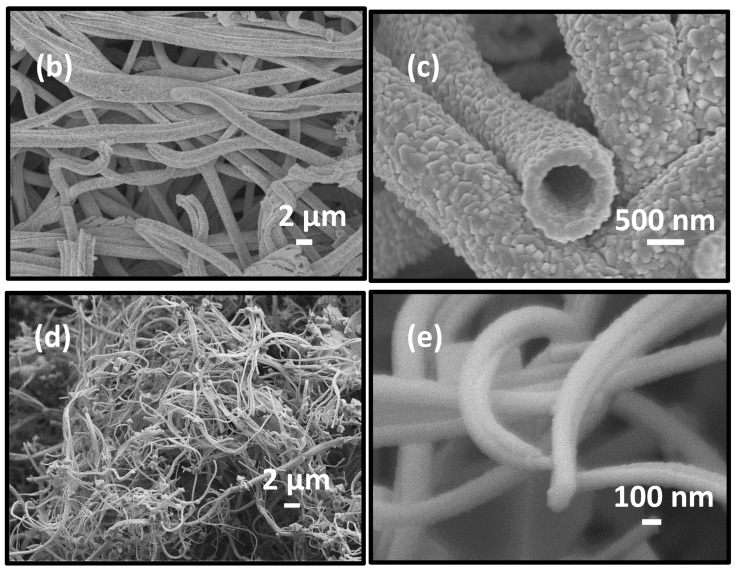
FESEM images of (**a**) 9.0 (w/w)% ITO precursor loaded PVP-InCl_3_-SnCl_4_ salt precursor fibers (Inset: Image at higher magnification); (**b**,**c**) corresponding ITO nanofibers calcined at 700 °C for 2 h with a heating rate of 5 °C min^−1^; and (**d**,**e**) corresponding ITO nanofibers calcined at 700 °C for 2 h with a heating rate of 0.5 °C min^−1^.

The polymer fibers containing PVP and precursor salts InCl_3_ and SnCl_4_ were of solid cylindrical shape with smooth surfaces (see Figure 2a) and with diameter distributed in the range of ca.~700–1500 nm. The calcined fibers showed rough surfaces and reduced diameter (Figure 2b–e) due to loss of PVP in the calcination step. 

The ITO fibers obtained after calcination at a heating rate of 5 °C min^−1^ show fibrous morphology (Figure 2b) with diameter ca.~600–1200 nm and several tens of micrometer in length. The cross-section of a representative broken nanofiber shown in Figure 2c presents evidence that ITO nanofibers produced at 5 °C min^−1^ heating rate were hollow. The ITO nanofibers prepared at slower heating rate of 0.5 °C min^−1^ show significant reduction of diameter compared to the precursor nanofibers, e.g., diameter fell in the range of 50–650 nm with an average diameter of 335 ± 155 nm (see Figure 2d,e and Figure S1).

**Figure 3:**
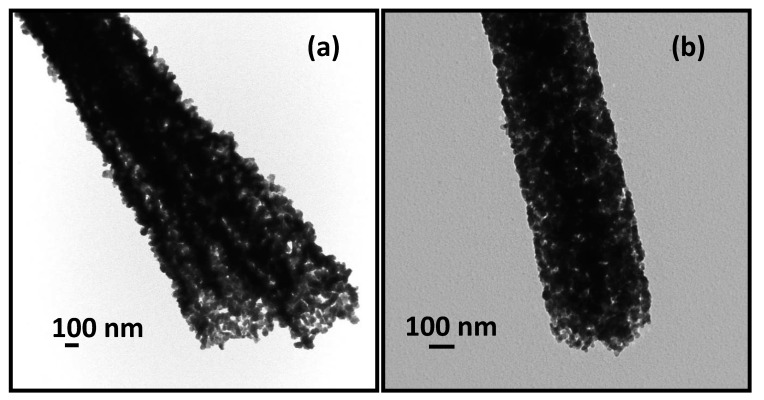
TEM images of ITO nanofiber calcined at 700 °C for 2 h with heating rates of (**a**) 5 °C min^−1^ and (**b**) 0.5 °C min^−1^ obtained from 9.0 (w/w)% ITO precursor loaded spinning solution.

The porous nature of ITO nanofibers is evident from the TEM images presented in Figure 3. The TEM image in Figure 3a shows ITO grains of size ca.~60–70 nm organized around an annulus corroborating hollow structures as seen in SEM image in Figure 2c. The TEM image in Figure 3b of ITO nanofibers obtained at slower heating rate of 0.5 °C min^−1^ shows porous, but non-hollow structures formed by finer grains of ITO crystals of size ca.~20–30 nm. The images in Figure 3 indicate that high heating rate caused rapid growth of crystal nucleus to form large size ITO grains whereas smaller ITO grains resulted from slower heating rate. 

**Figure 4:**
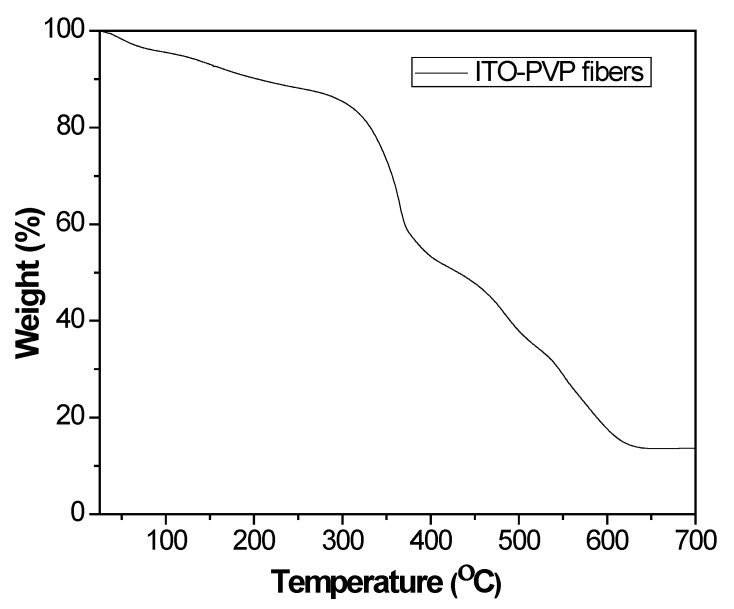
TGA trace of thermal decomposition of as-spun PVP-InCl_3_-SnCl_4_ salt precursor fibers obtained from a 9.0 (w/w)% ITO precursor loaded spinning solution in air.

The stages of thermal degradation of precursor fibers containing InCl_3_ and SnCl_4_ salt were monitored using TGA under air, although a different heating rate, 10 °C min^−1^ was used. The weight vs. temperature trace presented in Figure 4 show that the entire thermal degradation process consists of three weight loss events. The first slow weight loss event occurred up to 250 °C due to degradation of the side chain of PVP and evaporation of the trace amounts of entrapped ethanol, DMF, and small amount of moisture in precursor polymer fibers [7,21]. Approximately 5.0 wt % loss during heating to 100 °C can be attributed to the loss of moisture and solvents ethanol and DMF. Subsequent weight loss was due to degradation of side chains of PVP. The obvious weight loss between 250 °C and 400 °C can be attributed to decomposition by oxidation of the main chain PVP polymer and loss of water from the hydrolysis products of InCl_3_ and SnCl_4_. Additional weight reduction between 450 °C and 600 °C occurred due to decomposition of residual polymer mass. The weight was almost constant at above 600 °C, indicating complete transformation of polymer precursor nanofibers into ITO nanofibers [21,23]. 

**Figure 5:**
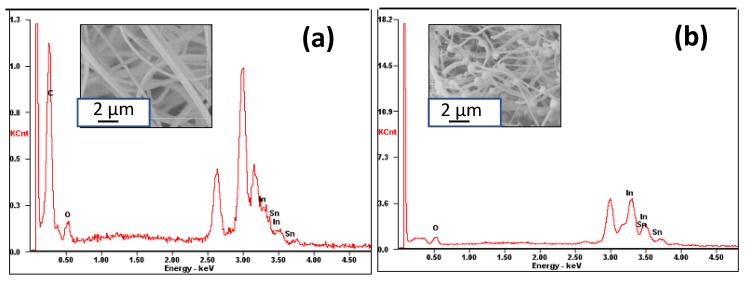
EDS pattern of (**a**) polymer fibers loaded with 9.0 (w/w)% of InCl_3_ and SnCl_4_, and (**b**) corresponding ITO nanofibers calcined at 700 °C with a heating rate of 0.5 °C min^−1^.

A comparison of EDS data presented in Figure 5a,b and Table S1 reveals that ITO nanofibers were composed entirely of In, Sn, and O elements without any impurity while precursor polymer fibers contained significant fraction of carbon due to high content of PVP. The atomic and weight percent of individual elements in each sample are presented in the inset of Figure 5. The absence of peak due to carbon in calcined fibers indicates complete removal of PVP in the calcination step. The appearance of intense peak at ~3.0 eV in Figure 5a,b is due to elemental silver (Ag) used in sputter coating the fiber samples before SEM characterization. 

**Figure 6:**
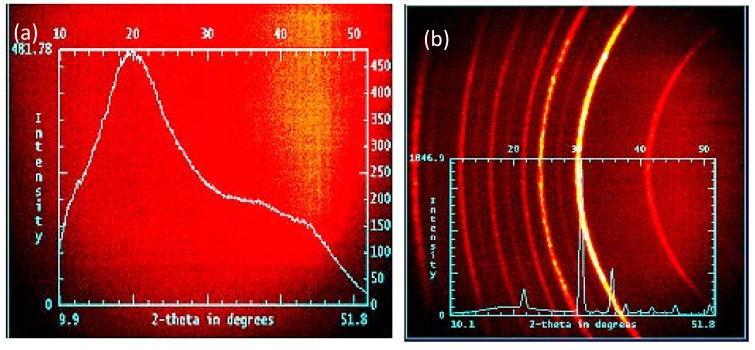
WAXD Patterns of (**a**) 9.0 (w/w)% ITO precursor loaded PVP-InCl_3_-SnCl_4_ salt precursor fibers, and (**b**) corresponding ITO nanofibers calcined at 700 °C with a heating rate of 0.5 °C min^−1^.

The information on crystal structure of the calcined fibers were gleaned from wide angle X-ray diffraction (WAXD) data presented in Figure 6. The WAXD spectra of PVP-InCl_3_-SnCl_4_ salt precursor fibers show amorphous characteristic attributed to the presence of large amounts of PVP (see Figure 6a) [38]. The WAXD data of ITO fibers (Figure 6b) indicate that these fibers were composed of In_2_O_3_ body centered cubic crystals as evident from a strong peak at 2θ = 30.6° [23].

The measured WAXD pattern exhibits diffraction peaks at (211), (222), (400), (411), (332), (431), and (440) planes, which is in agreement with JCPDS card no. 6–0416 for cubic crystal structure of In_2_O_3_ [32].However, the major peaks due to SnO and SnO_2_ expected to appear at 2θ = 33.2°, 26.5°, and 35.5° are absent in the WAXD pattern. These imply that tin (Sn) atoms were probably doped into the In_2_O_3_ crystal [36,38].We note that Sn is a tetravalent element and its incorporation in ITO structure via doping of In_2_O_3_ crystals means substitution of one In^3+^ from In_2_O_3_ crystal lattice position by one Sn^4+^ ion, thereby donating a free electron for conductivity in ITO material. The crystallite size of the ITO nanofiber calcined at 700 °C is ca. 14 nm as calculated from WAXD data using Scherrer’s equation [26]. The calculated crystallite size is close to the grain size inferred from the TEM image of the ITO nanofiber in Figure 3b.

### 3.2. Morphology of ITO: Effect of ITO Precursor Concentration 

As discussed up to this point, the representative ITO fibers obtained from calcination of precursor polymer fibers containing 9 (w/w)% InCl_3_ and SnCl_4_ at 700 °C showed two different morphological forms – (i) free standing, highly crystalline, and porous non-hollow ITO nanofibers at slow heating rate (0.5 °C min^−1^) and (ii) free standing, highly crystalline, porous hollow structure of ITO produced at high heating rate (5 °C min^−1^). We recognize that the porous morphology of semiconducting metal oxide nanofibers such as TiO_2_ and V_2_O_5_ are beneficial in efficient photocatalytic oxidation of volatile organic compounds (VOCs) as reported in our earlier work [6,26]. Faster diffusion of the gas molecules and availability of higher surface area active materials in the porous fibers enhance the photocatalytic oxidation reactions. In this regard, the strategy of producing porous hollow SMO fibers can be implemented for photocatalyst materials. 

**Figure 7:**
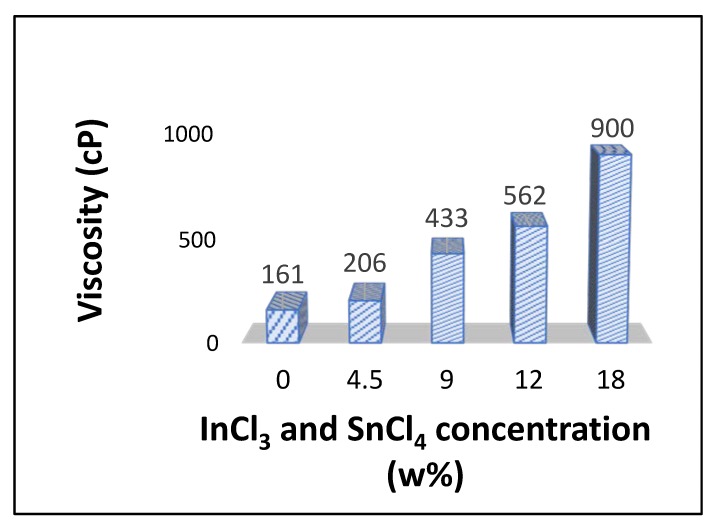
Steady state shear viscosity of PVP solutions at different concentrations of InCl_3_ and SnCl_4_ at 9:1 by weight ration of In and Sn.

It is important to determine if the heating rate and the salt concentration in precursor fibers also influenced ITO fiber morphology. An immediate effect of an increase of InCl_3_ and SnCl_4_ concentration in the spinning solution is significant increase of viscosity as shown in Figure 7.

The data in Figure 7 indicate substantial increase of shear viscosity with the addition of ITO precursor salts, e.g., shear viscosity increased from 161 cP for PVP solution with no inorganic salts to 206 cP for 4.5 (w/w)% ITO precursor loading and finally to 900 cP with 18 (w/w)% precursor salts. The viscosity of the spinning solution at 9 (w/w)% precursor salt concentration was 433 cP, almost twice as much of the solution with 4.5 (w/w)% salt and less than half of the solution with 18 (w/w) % salt. We attribute this increase of viscosity in the presence of precursor salts to formation of complex structures between PVP and indium and tin ions [39]. We note that an increase of spinning solution viscosity results in an increase of diameter of polymer precursor fibers produced by GJF process [40]. In GJF process, a liquid jet produced from spinning solution of higher viscosity undergoes lesser stretching at a given air pressure [22].

A set of representative images of polymer precursor fibers produced at different salt concentration are shown in Figure S2. The precursor fibers corresponding to 4.5 (w/w)% salt appear smooth (Figure S2a). However, the spinning solutions with 12 and 18 (w/w)% salt produced fibers with significant amounts of beads (Figure S2c,d). The non-beaded fraction of fibers in these cases were of diameter as large as 5 µm.

**Figure 8:**
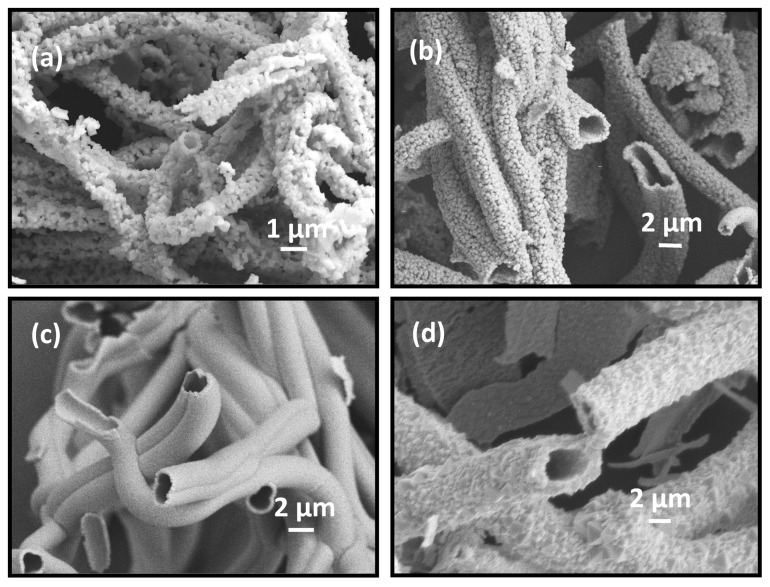
FESEM images of morphologies of ITO fibers obtained by calcining at 700 °C for 2 h with a heating rate of 5 °C min^−1^ obtained from InCl_3_ and SnCl_4_ concentration of (**a**) 4.5, (**b**) 9.0, (**c**) 12.0, and (**d**) 18.0 (w/w)%.

**Figure 9:**
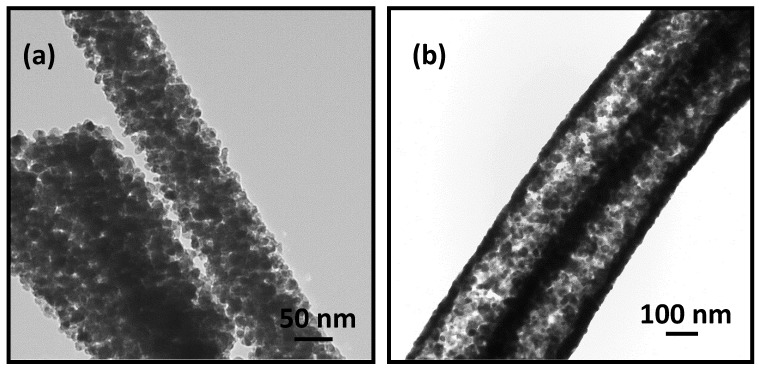
TEM images of ITO fibers obtained by calcining at 700 °C for 2 h with heating rates of (**a**) 0.5 and (**b**) 5 °C min^−1^ from 4.5 (w/w)% solution of InCl_3_ and SnCl_4_ in solution with PVP.

The ITO fibers obtained at higher heating rate (5 °C min^−1^) were hollow, irrespective of the precursor salt concentration in the spinning solutions, as shown in Figure 8. However, the ITO shells appear highly porous for fibers produced with 4.5 (Figure 8a) and 9 (w/w)% salt concentration (Figure 8b). The ITO shells in fibers corresponding to 12 and 18 (w/w) % salt concentration appear much denser and thicker, leading to a microtubular morphology (Figure 8c,d and Table 1). The images in Figure 8 indicate that hollow ITO fibers and microtubes had diameter in the range of 800 nm–4.0 µm depending on the precursors salt concentration in the spinning solution. As shown in Figure 8a, smaller diameter hollow ITO fibers with diameter 800–1500 nm were obtained at lower salt concentration of 4.5 (w/w)%. The ITO fibers produced from the same set of spinning solutions, but calcined at a slower heating rate of 0.5 °C min^−1^, exhibited non-hollow porous cylindrical morphology as seen earlier in Figure 2e and Figure 3b for 9 (w/w)% salt solutions. As reported in our previous work, we observed similar non-hollow porous cylindrical morphology of TiO_2_ nanofibers with a heating rate of 0.5 °C min^−1^ [26]. A comparison of TEM images in Figure 9 of ITO fibers produced from 4.5 (w/w)% salt solution also establishes that a slower heating rate of 0.5 °C min^−1^ helped produce non-hollow ITO fibers (Figure 9a) while hollow fibers with an empty core was produced at a heating rate of 5 °C min^−1^ as evident from the TEM image presented in Figure 9b. The fiber diameter produced at slower heating rate fell in the range of ~150–400 nm with an average diameter of ca. 320 ± 80 nm. 

### 3.3. Formation Mechanism of ITO Fibers 

Based on the above experimental observations, we propose a mechanism for formation of different morphological forms of ITO fibers. We observed and discussed earlier three distinct morphological forms of ITO fibers – (i) porous solid cylindrical fibers, (ii) porous, hollow cylindrical fibers, and (iii) hollow, microtubular fibers obtained by varying the concentration of precursor salt in the spinning solution and the heating rate in the calcination step. Hollow ITO fibers were observed at high heating rates (5 °C min^−1^ or greater), irrespective of the salt concentration in the precursor solution (see Figure 2b,c and Figure 8). We attribute this to faster rise of gas pressure at the center of the polymer precursor fibers at the onset of decomposition of carrier polymer PVP. In calcination step, PVP decomposes and degrades into gaseous substances, such as CO_2,_ NO_2_, and H_2_O [23,26]. These gases generate a pressure difference between the center and the outer domains of the nanofibers [41]. The gas evolved from the faster degradation of the polymer at or near the center of the fiber accumulates and pushes the solid domains outward. This leads to hollow structure of ITO fibers after complete decomposition of PVP [2,7]. When the calcination step was carried out at 10 °C min^−1^ or greater heating rate, only broken and irregular tubular structures were obtained (see Figure S3). This illustrates that tubular structures of ITO fibers could not withstand the internal pressure generation by the gas evolving in calcination step. Another reason may be the high thermal stress resulting from the quick heating during calcination process. A similar observation was reported for electrospun TiO_2_ and Fe_2_O_3_ micro tubular structures elsewhere [7]. At a slower heating rate, e.g., 0.5 °C min^−1^, the top surface layer and sub-layer of the precursor polymer fibers melted and produced degradation gases easily. This results in the formation of non-hollow structure of ITO fibers with either porous (in case of lower ITO concentration in the precursor solution) or compact (for higher ITO concentration) surface after complete degradation of the PVP (See Figure 2c,d and Figure 9a). Although the data presented in this paper support the above mechanism, we believe that a detailed study is needed to understand further the mechanism of such micro and nanostructure formation, especially in relation to other types of SMOs.

## 4. Conclusions

We report for the first time a one-step fabrication strategy for fabrication of ITO porous nanofibers and microtubes with porous or solid shells formed by the crystalline grains of ITO. The polymer precursor fibers obtained by gas jet spinning from solutions of PVP, InCl_3_, and SnCl_4_ showed smooth fiber surfaces. However, the loss of carrier polymer PVP during calcination process yielded several morphological forms as function of heating rate in the calcination step. At a high heating rate of 5 °C min^−1^, hollow ITO tubular structures were formed irrespective of salt concentration in the spinning solutions. The shell thickness of the tubular structure, however, shows strong dependence on salt concentration, e.g., porous shell at lower salt concentration of 4.5 and 9.0 (w/w)% while solid shell at higher concentration of 18.0 (w/w)%. At slower heating rate, 0.5 °C min^−1^, however, non-hollow porous morphologies were obtained irrespective of salt concentration in the spinning solutions. The strategy of using heating rate during calcination process may promote fabrication of hollow tubular and non-hollow fibrous structures for other inorganic oxide materials.

## Figures and Tables

**Table 1:** Effect of ITO precursor concentration and heating rate on morphology of ITO structures.

Sample	Morphology after Calcination at 700 °C with Heating Rates of	
	Lower (0.5 °C min^−1^)	Higher (5.0 °C min^−1^)
4.5 w% ITO precursor loaded PVP solution	Porous cylindrical nanofiber	Highly porous hollow fiber
9.0 w% ITO precursor loaded PVP solution	Compact cylindrical nanofiber	Porous hollow fiber
12.0 w% ITO precursor loaded PVP solution	Solid fiber with traces of agglomeration	Thin walled hollow microtube
18.0 w% ITO precursor loaded PVP solution	-	Thick walled hollow microtube and agglomerated mass

## Supplementary Materials

The following are available online at https://www.mdpi.com/1996-1944/13/7/1539/s1, Figure S1: Diameter distribution of ITO nanofibers calcined at 700 °C with a heating rate of 0.5 °C min^−1^, Figure S2: FESEM images of polymer precursor fibers obtained from a PVP-InCl_3_-SnCl_4_ spinning solution containing different (w/w)% of ITO precursor concentration (a) 4.5, (b) 9.0, (c) 12.0, and (d) 18.0. All experiments were performed under identical conditions as mentioned in the experimental section, Figure S3: FESEM images of calcined fibers obtained with 9.0 (w/w)% ITO precursor salts in the spinning solution. Fibers were calcined at 700 °C for 2 h with a heating rate of >10 °C min^−1^. Images at (a) low magnification, and (b) high magnification, Table S1: Elemental compositional analysis of (a) polymer fibers loaded with 9.0 (w/w)% of InCl_3_ and SnCl_4_, and (b) corresponding ITO nanofibers calcined at 700 °C with a heating rate of 0.5 °C min^−1^.

## References

[1] Yoon S., Kim H., Shin E.-S., Huh J.N., Noh Y.-Y., Park B., Hwang I. (2017). Toward High Conductivity of Electrospun Indium Tin Oxide Nanofibers with Fiber Morphology Dependent Surface Coverage: Postannealing and Polymer Ratio Effects. ACS Appl. Mater. Interfaces.

[2] Lang L., Wu D., Xu Z. (2012). Controllable Fabrication of TiO_2_ 1D-Nano/Micro Structures: Solid, Hollow, and Tube-in-Tube Fibers by Electrospinning and the Photocatalytic Performance. Chem. Eur. J..

[3] Li D., Wang Y., Xia Y. (2003). Electrospinning of Polymeric and Ceramic Nanofibers as Uniaxially Aligned Arrays. Nano Lett..

[4] Mitra J., Ghosh M., Bordia R.K., Sharma A. (2013). Photoluminescent Electrospun Submicron Fibers Of Hybrid Organosiloxane And Derived Silica. RSC Adv..

[5] Xu S., Shi Y. (2009). Low Temperature High Sensor Response Nano Gas Sensor Using ITO Nanofibers. Sens. Actuators B Chem..

[6] Ghosh M., Liu J., Chuang S.S.C., Jana S.C. (2018). Fabrication of Hierarchical V_2_O_5_ Nanorods on TiO_2_ Nanofibers and Their Enhanced Photocatalytic Activity under Visible Light. ChemCatChem.

[7] Lang L., Xu Z. (2013). Controllable Synthesis of Porous α-Fe_2_O_3_ Microtube and Tube-in-tube by Non-coaxial Electrospinning. Chem. Lett..

[8] Wan Q., Wei M., Zhi D., MacManus-Driscoll J.L., Blamire M.G. (2006). Epitaxial Growth of Vertically Aligned and Branched Single-Crystalline Tin-Doped Indium Oxide Nanowire Arrays. Adv. Mater..

[9] Almasian A., Chizari Fard G., Maleknia L. (2017). Fabrication Of Hollow And Nonhollow Sio2 Nanofibers For Removal of Cationic Dyes From Aqueous Solutions. Environ. Progress Sustain. Energy.

[10] Wang L., Luo X., Zheng X., Wang R., Zhang T. (2013). Direct Annealing Of Electrospun Synthesized High-Performance Porous Sno2 Hollow Nanofibers For Gas Sensors. RSC Adv..

[11] Zhang Z., Li X., Wang C., Wei L., Liu Y., Shao C. (2009). ZnO Hollow Nanofibers: Fabrication from Facile Single Capillary Electrospinning and Applications in Gas Sensors. J. Phys. Chem. C.

[12] Cheng J.P., Wang B.B., Zhao M.G., Liu F., Zhang X.B. (2014). Nickel-Doped Tin Oxide Hollow Nanofibers Prepared By Electrospinning For Acetone Sensing. Sens. Actuators B Chem..

[13] Einert M., Ostermann R., Weller T., Zellmer S., Garnweitner G., Smarsly B.M., Marschall R. (2016). Hollow A-Fe_2_O_3_ Nanofibres for Solar Water Oxidation: Improving The Photoelectrochemical Performance By Formation of A-Fe_2_O_3_/ITO-Composite Photoanodes. J. Mater. Chem. A.

[14] Zhang X., Thavasi V., Mhaisalkar S.G., Ramakrishna S. (2012). Novel Hollow Mesoporous 1D Tio2 Nanofibers As Photovoltaic And Photocatalytic Materials. Nanoscale.

[15] Mao X., Si Y., CHen Y., Yang L., Zhao F., DIng B., Yu J. (2012). Silica Nanofibrous Membranes With Robust Flexibility And Thermal Stability For High-Efficiency Fine Particulate Filtration. RSC Adv..

[16] Lakshmi B., Patrissi C., Martin C. (1997). Sol−Gel Template Synthesis of Semiconductor Oxide Micro- And Nanostructures. Chem. Mater..

[17] Li D., McCann J.T., Xia Y. (2005). Use of Electrospinning to Directly Fabricate Hollow Nanofibers with Functionalized Inner and Outer Surfaces. Small.

[18] Srivastava Y., Loscertales I., Marquez M., Thorsen T. (2008). Electrospinning of Hollow And Core/Sheath Nanofibers Using A Microfluidic Manifold. Microfluid. Nanofluidics.

[19] Yasuda K., Schmuki P. (2007). Formation of Self-organized Zirconium Titanate Nanotube Layers by Alloy Anodization. Adv. Mater..

[20] Jia C.J., Sun L.D., Yan Z.G., You L.P., Luo F., Han X.D., Pang Y.C., Zhang Z., Yan C.H. (2005). Single-Crystalline Iron Oxide Nanotubes. Angew. Chem..

[21] Wu J., Zeng D., Wang X., Zeng L., Huang Q., Tang G., Xie C. (2014). mechanistic Insights Into Formation Of Sno2 Nanotubes: Asynchronous Decomposition Of Poly(Vinylpyrrolidone) In Electrospun Fibers During Calcining Process. Langmuir.

[22] Benavides R.E., Jana S.C., Reneker D.H. (2012). Nanofibers from Scalable Gas Jet Process. ACS Macro Lett..

[23] Ghosh M., Jana S.C. (2015). Bi-Component Inorganic Oxide Nanofibers from Gas Jet Fiber Spinning Process. RSC Advances.

[24] Rajgarhia S.S., Jana S.C. (2016). Comparison of Electrospinning and Gas Jet Fiber Processes for Fabrication of Bi-Component Polymer Nanofibers from Single Solutions. Macromol. Symp..

[25] Ghosh M., Jana S.C. (2016). Fabrication, Morphological Evaluation, and Characterization of Semiconducting Oxide Nanofibers from Gas Jet Fiber Spinning Process. Proceedings of the ANTEC 2016.

[26] Ghosh M., Lohrasbi M., Chuang S.S.C., Jana S.C. (2016). Mesoporous Titanium Dioxide Nanofibers with a Significantly Enhanced Photocatalytic Activity. ChemCatChem.

[27] O’Dwyer C., Szachowicz M., Visimberga G., Lavayen V., Newcomb S.B., Torres C.M.S. (2009). Bottom-Up Growth Of Fully Transparent Contact Layers Of Indium Tin Oxide Nanowires for Light-Emitting Devices. Nat. Nanotechnol..

[28] Iskandar F., Suryamas A.B., Kawabe M., Munir M.M., Okuyama K., Tarao T., Nishitani T. (2010). Indium Tin Oxide Nanofiber Film Electrode for High Performance Dye Sensitized Solar Cells. Jpn. J. Appl. Phys..

[29] Hong-Wen W., Chi-Feng T., Miao-Ken H., Chwei-Huann C., Ying-Ling L., Zongwen L., Kyle R.R., Simon P.R. (2009). Three-Dimensional Electrodes For Dye-Sensitized Solar Cells: Synthesis Of Indium–Tin-Oxide Nanowire Arrays And ITO/TiO_2_ Core–Shell Nanowire Arrays By Electrophoretic Deposition. Nanotechnology.

[30] Chuangchote S., Sagawa T., Yoshikawa S. (2011). Indium Tin Oxide Nanofibers and their Applications for Dye-Sensitized Solar Cells. ECS Trans..

[31] Lee D., Kim B., Kim J., Jeong S., Cao G., Moon J. (2015). Salami-like Electrospun Si Nanoparticle-ITO Composite Nanofibers with Internal Conductive Pathways for use as Anodes for Li-Ion Batteries. ACS Appl. Mater. Interfaces.

[32] Jin M.-J., Ma T., Ling T., Qiao S.-Z., Du X.-W. (2012). Three-Dimensional Networks Of ITO/Cds Coaxial Nanofibers For Photovoltaic Applications. J. Mater. Chem..

[33] Luff B.J., Wilkinson J.S., Perrone G. (1997). Indium Tin Oxide Overlayered Waveguides for Sensor Applications. Appl. Opt..

[34] Wu H., Hu L., Carney T., Ruan Z., Kong D., Yu Z., Yao Y., Cha J.J., Zhu J., Fan S. (2011). Low Reflectivity and High Flexibility of Tin-Doped Indium Oxide Nanofiber Transparent Electrodes. J. Am. Chem. Soc..

[35] Kim C., Choi W., Cho S., Goshima D., Ham D., Kim K., Jeong J., Lee J., Lee S. (2015). Fabrication of Structurally Simple Index-Matched ITO Films Using Roll-to-Roll Sputtering for Touch Screen Panel Devices. Plasma Process. Polym..

[36] Munir M.M., Widiyandari H., Iskandar F., Okuyama K. (2008). Patterned Indium Tin Oxide Nanofiber Films and Their Electrical and Optical Performance. Nanotechnology.

[37] Mukhlish M.Z.B., Horie Y., Higashi K., Ichigi A., Guo S., Nomiyama T. (2017). Self-Standing Conductive ITO-Silica Nanofiber Mats for Use In Flexible Electronics And Their Application In Dye-Sensitized Solar Cells. Ceram. Int..

[38] Muhammad Miftahul M., Ferry I., Ki Myoung Y., Kikuo O., Mikrajuddin A. (2008). Optical And Electrical Properties of Indium Tin Oxide Nanofibers Prepared By Electrospinning. Nanotechnology.

[39] Jiang H., Moon K.-S., Sun Y., Wong C.P., Hua F., Pal T., Pal A. (2008). Tin/Indium Nanobundle Formation from Aggregation Or Growth Of Nanoparticles. J. Nanoparticle Res..

[40] Reneker D.H., Yarin A.L. (2008). Electrospinning Jets and Polymer Nanofibers. Polymer.

[41] Cheng Y., Zou B., Yang J., Wang C., Liu Y., Fan X., Zhu L., Wang Y., Ma H., Cao X. (2011). Fabrication Of CoFe_2_O_4_ Hollow Fibers By Direct Annealing of The Electrospun Composite Fibers And Their Magnetic Properties. CrystEngComm.

