# Supplementary material for: Fabrication of Hollow and Porous Tin-Doped Indium Oxide Nanofibers and Microtubes via a Gas Jet Fiber Spinning Process
Source: Materials (Basel). 2020 Mar 27;13(7):1539. doi: 10.3390/ma13071539 (PMC7177504; doi:10.3390/ma13071539)
Supplement: Supplementary file 1 [file materials-13-01539-s001.pdf]

materials

Article

Fabrication of Hollow and Porous Tin-Doped Indium Oxide Nanofibers and Microtubes via a Gas Jet Fiber Spinning Process

Monoj Ghosh and Sadhan C. Jana *

Department of Polymer Engineering, The University of Akron, 250, South Forge Street, Akron, 44325–0301, OH, USA; mg84@zips.uakron.edu

* Correspondence: janas@uakron.edu; Tel.: +1–330–972–8293

Received: 10 March 2020; Accepted: 25 March 2020; Published: date

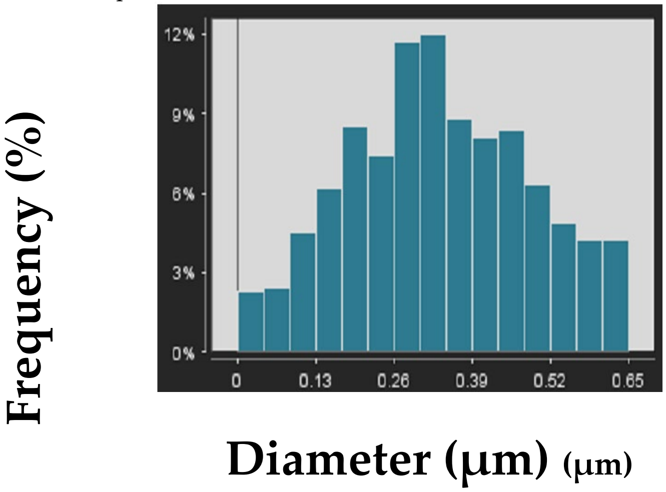

Figure S1. Diameter distribution of ITO nanofibers calcined at 700 °C with a heating rate of 0.5 °C min^{-1}.

materials

MDPI

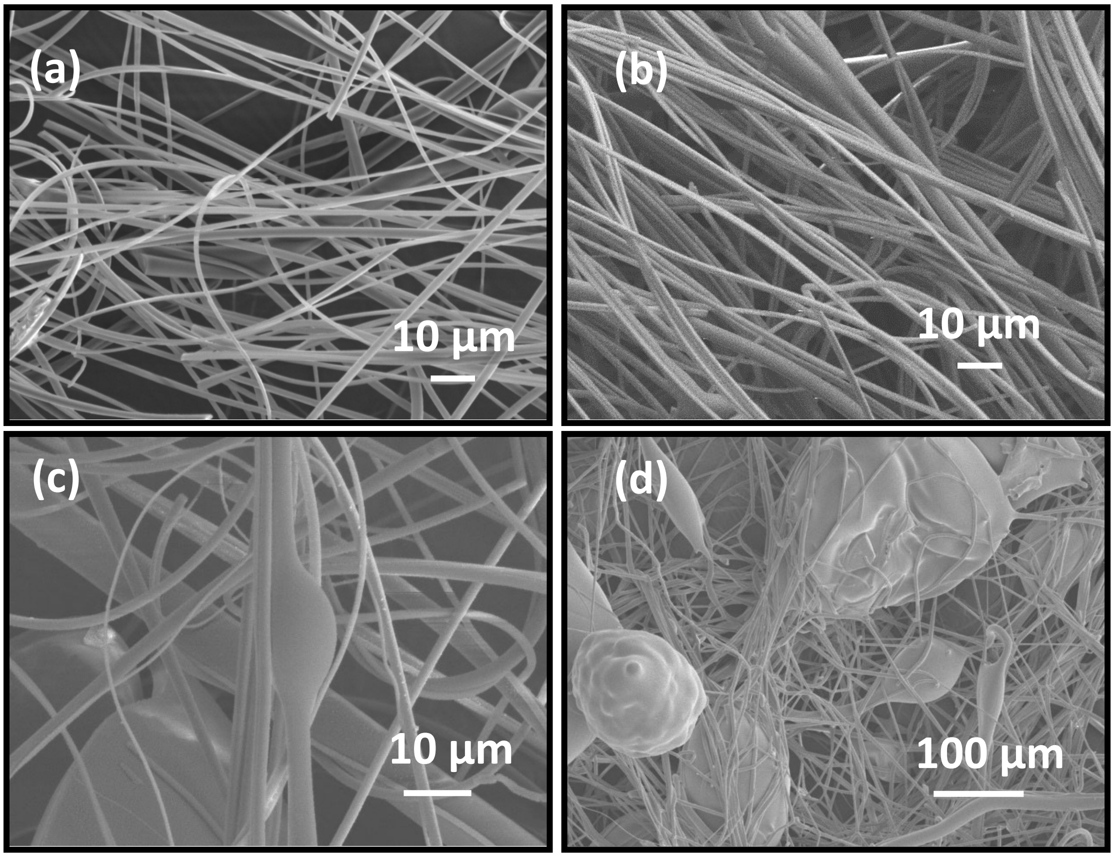

Figure S2. FESEM images of polymer precursor fibers obtained from a PVP-$InCl_3$-$SnCl_4$ spinning sol solution containing different (w/w) % of ITO precursor concentration (**a**) 4.5, (**b**) 9.0, (**c**) 12.0, and (**d**) 18.0. All experiments were performed under identical conditions as mentioned the experimental section.

GJF spinning process was not smooth, and tip of the needle clogged frequently due to higher viscosities of spinning solutions containing 12.0 and 18.0 (w/w) % ITO precursor with viscosities ~562 and ~900 cP, respectively. As a result, they produced fibers with agglomerated mass with a size in micrometers range.

materials

MDPI

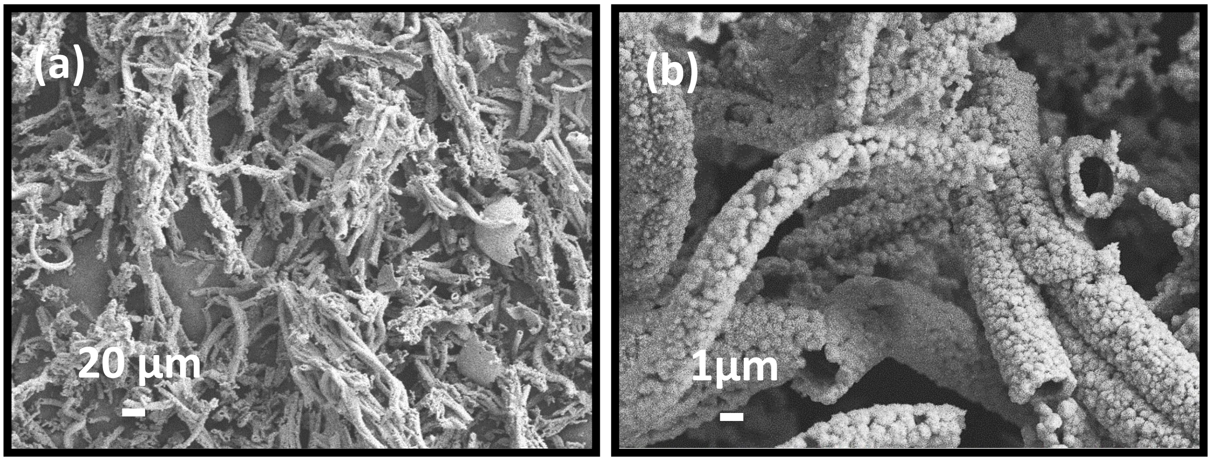

Figure S3. FESEM images of calcined fibers obtained with 9.0(w/w) % ITO precursor salts in the spinning solution. Fibers were calcined at 700 °C for 2 h with a heating rate of >10 °C min^{-1}. Images at (**a**) low magnification, and (**b**) high magnification.

Table 1. Elemental compositional analysis of (a) polymer fibers loaded with 9.0 (w/w) % of $InCl_3$ and. $SnCl_4$, and (b) corresponding ITO nanofibers calcined at 700 °C with a heating rate of 0.5 °C min^{-1}.

(a) Polymer precursor fibers

Element	Weight%	Atom%
**CK**	53.57	80.47
**OK**	12.61	14.22
**InL**	33.02	05.19
**SnL**	00.80	00.12

(b) ITO nanofibers

Element	Weight%	Atom%
**OK**	14.20	54.31
**InL**	84.74	45.15
**SnL**	0.06	00.54
